# A Biomorphic Approach to Designing Special-Purpose Vehicles for Arctic Conditions

**DOI:** 10.3390/biomimetics8040360

**Published:** 2023-08-11

**Authors:** Nikita Klyusov, Nikolai Garin, Svetlana Usenyuk-Kravchuk, Ekaterina Vasilieva, Kirill Ustinov

**Affiliations:** 1 Arctic Design Lab, Ural Federal University, Yekaterinburg 620000, Russia; 2 Industrial Design Department, Ural State University of Architecture and Art, Yekaterinburg 620075, Russia

**Keywords:** extreme environment, context-based design, vehicle design, biomimetics, biomorphic approach

## Abstract

The paper explores the potential of the biomorphic approach to context-based design with a focus on special-purpose mobility in the Arctic. The study seeks to contribute to the analytical and conceptual basis for developing the transport component of the Arctic life-support system, i.e., a set of objects and technologies, and knowledge and skills for handling them, allowing a person to survive and comfortably exist in severe environmental conditions. The central argument is that the system should incorporate structural components that possess not only technical but also artistic and emotional characteristics that align with the geographic (environmental and climatic), socio-cultural, and psychological peculiarities of use. This can be achieved by drawing inspiration from local nature. We probe the visual image of “soft military presence” using two case studies in different parts of the Russian Arctic: the Yamal and Chukchi peninsulas.

## 1. Introduction

The trends observed in the two last decades in Arctic development [1,2,3,4], including escalating concerns over resources and boundaries, have resulted in an increase in international military presence in the region. Furthermore, the recent extension of NATO membership to include Finland and Sweden has already elevated the Arctic and the High North to a more central position in NATO’s strategy and posture [5]. 

In this paper, we study Russia’s “soft military presence” in the Arctic through the lens of design, drawing on Russia’s views of the issues and opportunities offered by the changing and challenging Arctic environment, and considering how Russia seeks to balance national economic, environmental, and security interests in the region. This focus is important for understanding the country’s approach to security and cooperation with other Arctic nations.

The term “soft military presence” (or footprint) coined by Degang Sun [6,7] means the priority of mission-oriented tasks and the prevalence of the public good over geopolitical rivalry. In our paper, we use this term with reference to Russia’s border forces in the Arctic. While Russia’s Arctic strategy for 2020-35 prioritizes building a military presence to protect the country’s Arctic territory and maritime space [8], these forces are oriented towards a “soft military footprint” to reflect the country’s position that the challenges facing the Arctic region are not of a military nature and do not (yet) require hard military solutions. Within this priority, the Arctic zone benefits from Russia’s military build-up as a concerted state effort to develop the region by implementing technological solutions that have both civilian and military applications [9].

The border forces in the Arctic region are responsible for a range of tasks beyond military applications. These include search and rescue operations, countering terrorism and illegal migration, ensuring the safety of maritime transport along the Northern Sea Route and the security of its marine and terrestrial infrastructure, monitoring fishing activities in the northern latitudes, contributing to the development of resources on the Arctic continental shelf, protecting the environment, and accomplishing other military and civilian missions [10]. We focus on the visual image of “soft military presence” and probe it by engaging students in the exploration of the biomorphic approach to the context-based design of Arctic special-purpose vehicles considering the international trend of Arctic militarization. There is a growing emphasis on special means to facilitate human presence in the extreme environment: first and foremost, economically and environmentally efficient solutions to the transportation problem ensure uninterrupted all-weather mobility [11]. In the case of Russia, today’s developments are largely moving away from the practices of its Soviet forebears, who preferred standardization with variations on existing frames rather than niche production with new designs altogether [12]. 

The paper represents a special direction in the design theory and practices pursued at the Arctic Design School, i.e., the design of life-support systems for an extreme environment [13] focusing on the military sector. The purpose of this study was to prepare an analytical and conceptual basis for developing the transport component of the Arctic life-support system, i.e., a set of objects and technologies, as well as knowledge and skills needed for handling them, allowing a person to survive and comfortably exist in extreme environmental conditions. Regarding military applications, we argue that while ensuring the safety and maintaining the functional readiness of a serviceman, the system should feature a certain set of not only technical but also artistic and emotional characteristics corresponding to the environmental/climatic, socio-cultural, and psychological peculiarities of use. We probe the visual image of “soft military presence” in two case studies of student projects of transport vehicles for different parts of the Russian Arctic: the Yamal and Chukchi peninsulas. 

The article is structured as follows: first, we describe the data collected prior to carrying out the projects. Next, the working conditions in the selected study localities are summarized. Then, we describe the specific design tasks and the ideation process, which includes analysis of animal and human mobility systems originating in the Arctic. This is followed by a presentation of the generated design solutions that incorporate biomimetic design principles and are tailored to the transportation needs of the selected study localities. Finally, we discuss the results, draw conclusions on their potential for improving transportation in the Arctic, and outline potential directions for further research and development.

## 2. Data and Methods

Initial data collection and design explorations into the traditional vehicles and daily mobility of Arctic indigenous nomads were carried out by students and researchers of the Arctic Design School during expeditions to various localities of the Russian North in the late 1980s–early 1990s. While the ideas based on these data were incorporated into earlier students’ projects [14], the bionic design approach presented in this paper was first conceptualized and developed during these two projects and is being introduced for the first time.

The choice of the Yamal and Chukchi peninsulas was based on a combination of existing and potential military arrangements within Russia’s northern territories, as well as the availability of firsthand data. Accessing the Arctic is a challenging task that requires significant preparation and resources for fieldwork. Prior to the projects described in this paper, several field trips were conducted to Yamal and Chukotka, funded by the Ella and Georg Ehrnrooth Foundation and the Russian Science Foundation. These expeditions provided a wealth of qualitative and visual data on the working conditions, transportation needs, and existing transportation systems.

We collected firsthand data from the field and conducted a thorough review of contemporary transport technologies designed for military operations and civilian tasks in Arctic conditions. This included using open sources such as the internet, magazines, other mass media, and public exhibitions showcasing Arctic technologies. We employed context-based design [15] as the fundamental approach to designing for extreme environments, placing emphasis on what the Arctic environment affords and, eventually, what kind of design it fosters. Essentially, the experiment was based on biomimetics as a recognized method in ideation for gaining access to new and—for the designer—novel knowledge [16,17,18]. However, the stage of formulating and drawing generalized principles [19] was deliberately partly reduced to visual resemblance to living organisms, i.e., to a biomorphic approach that involves the use of organic shapes, patterns, and textures in design to create products that are aesthetically pleasing and functional. In developing artistic compositions that would give a nature-associated impression, we resorted to a biomorphic design to create products that are more sustainable, efficient, and user-friendly by mimicking natural systems and structures.

Finally, it is important to clarify that the projects did not aim to develop technical solutions based on the biology of Arctic animals. Instead, the team sought to grasp the integral image of successfully adapted living creatures with their specific locomotion and to use this understanding to inform the ideation and development of the transport component of the Arctic life-support system. While the construction, materials, and other technological adaptations are undoubtedly crucial to the final design, the team placed a particular emphasis on the concept and visual and intangible impression that the system should convey. By prioritizing the humanization and animation of technology, which is often overlooked due to the “harsh” character and purely utilitarian application of military vehicles and other equipment, the team hopes to contribute to a system that is not only functional and efficient but also aesthetically pleasing and emotionally engaging. 

## 3. Results

### 3.1. Working in the Arctic—Conditions and Constraints

The Arctic as a working environment presents a challenge to both humans coming from the middle and low latitudes and technology, particularly transport vehicles with chassis originally designed in and for a milder climate. Apparently, smooth human “integration” into the Arctic is impossible without transforming both the internal structure (physiology) and the psychological and social aspects, namely norms and values, habits, traditions, skills, and the surrounding material world [13]. As the Arctic design approach suggests, an immediate solution to the issue of adaptation to the extreme context would be a material environment that could complement and augment the physical capabilities of a user and have a positive effect on their psychological state.

**Figure 1:**
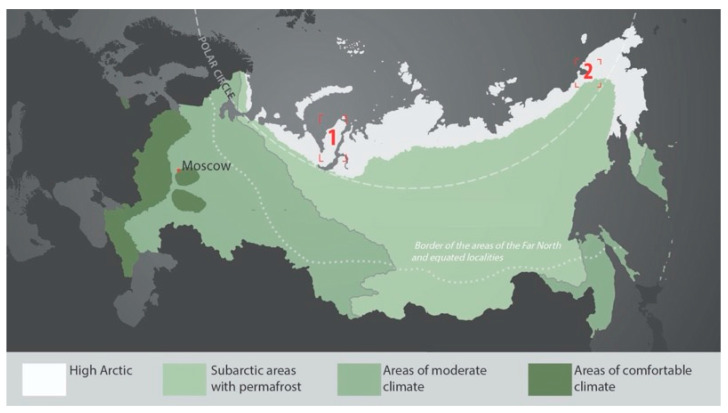
The geographical context of the study: 1—Yamal District; 2—Chaun District. Source: Authors.

Considering the existing and prospective military arrangements within Russia’s northern territories, we selected two regions for developing the proposed life support system, as follows: (1) Yamal Peninsula (Yamalsky District) and (2) Chukchi Peninsula (Chaunsky District) (Figure 1). The user is a serviceman, 20–45 years old, enlisted by contract, usually with a special-force background.

The basic set of Arctic natural and climatic constraints for outdoor work includes a difficult snow and ice situation, prolonged low temperatures, severe erosive effects of seawater, storms, strong gusty winds, poor visibility conditions, etc. 

The climate of the Yamal peninsula is primarily determined by the presence of permafrost and the cold Kara Sea, and the abundance of bays, rivers, marshes, and lakes. Winters are long (up to 8 months) with strong winds and thin snow cover (about 50 cm deep), whereas summers are short (about 50 days). The average annual air temperature is below 0 °C, reaching −10 °C in the Far North. The temperature in winter can drop to −60 °C, whereas in summer it can rise to +30 °C, with little precipitation of up to 400 mm per year. The surface of Yamal is mainly flat and low-lying, with a maximum height of 90 m. The coasts are sandy, and the inland area has an irregular surface that is significantly affected by marine and glacial deposition [20]. 

The Chaunsky District of Chukotka is flat compared to other districts in this autonomous okrug, with large expanses of reindeer pasturage. Wetland areas form a landscape that is almost entirely covered with small lakes. The climate is maritime, influenced by three neighboring seas: the Bering Sea, the East Siberian Sea, and the Chukchi Sea, which are characterized by cold northerly winds that can quickly change to wet southern winds. The average annual precipitation is between 200 and 400 mm per year. The temperature varies between −35 and −15 °C in January, and between +5 and +14 °C in July. Summer is short at only 80 to 100 days per year [21,22].

The adaptation of technology to the Arctic environment can be illustrated by the tracked military GT-T transporter, which is one of the most commonly used all-terrain vehicles in the region. Under Arctic conditions, the normal lifespan of special-purpose vehicles is significantly reduced (Table 1). To address this challenge, engineers utilize various special materials in the design of these vehicles, including cold-weather rubbers, plastics, fuels, lubricants, and metals. According to Dibbern [23], in the case of GT-T, these adaptations help to sustain the lifespan at only 6000 to 8000 km. These adjustments continue to be used for vehicles under Arctic conditions till today.

Having reviewed the transport systems of the selected regions and the specifics of military service, we identified the optimal transport unit as a small-sized vehicle for border patrolling. Thus, we propose a non-standard (special purpose) vehicle to fit the complex geographical and climatic features of the chosen regions, rather than using standard vehicles and equipment.

Arctic vehicles should not only withstand low temperatures but also easily overcome all kinds of roadlessness. Work in the coastal part of the northern seas requires machines to have amphibious properties, whereas operation in a swampy terrain requires all-terrain movement and stability [19]. Also, oversnow mobility sets more demanding requirements taking into account large variations and seasonality of the snow and ice cover [26,27,28].

The contextual sensitivity approach applied to transport vehicles is about matching the object designed to the geographical and climatic realities of the site of use and to the user, which leads us to the concept of a vehicle as an intermediary between the human and the environment and a guarantee of physical and psychological safety in the severe working conditions of the Arctic.

### 3.2. Design Development Process

A review of the existing types of terrestrial transport used in the northern roadlessness revealed that most of them do not meet the environmental and/or user requirements. According to our informants and recent studies (see [29,30,31]), most transport accidents in the North occur precisely due to insufficient information about the road and the terrain, and the overall stress experienced by a user/driver in severe climatic conditions. Therefore, the designers are faced with the challenge of rethinking the modes and means of mobility in this changing environment through more open and flexible vehicle design.

#### 3.2.1. Design Tasks

The project began by considering one of the primary design requirements for Arctic transport: any vehicle to be used in this extreme environment should be intuitively clear to operate. This implies that it is repairable in the field and easy to maintain using simple and widely available tools/equipment. Fancy novel “smart technologies” in such a vehicle may prove to be potentially dangerous as they require significant energy expenditures for use/maintenance, exacerbating potential risks in resource-constrained environments. 

For a small-size vehicle, it is equally important to develop an image-bearing figurative solution that establishes an emotional connection between the environment and the user. For a user, the vehicle should become more than a means of transportation; it should turn into a friend or even a protector. In the longer term, the lack of small-sized transports for the Far North on the mass market may be expected to cause military developments to expand into the civil sector and become generally available. Therefore, the initial user profile should be reconsidered from a broader perspective.

#### 3.2.2. The Ideation Process 

This section explores the potential of biomimetic design for Arctic transport by drawing inspiration from local analogues of mobility technology and know-how. The study begins with examining the life-sustaining systems of northern animals that are fully adapted to the evolving characteristics of the environment. It then delves into the mobility technologies and practices developed by Arctic indigenous peoples, whose material possessions and transport vehicles ingeniously imitate the surrounding living creatures. The goal of this section is to identify design features that can be applied to the development of sustainable and efficient transport vehicles for the Arctic.

### 3.3. The Art of Movement in the Arctic—How Animals Adapt to Their Environment

Studying the locomotion of arctic animals is crucial for designers as it provides valuable insights into how these animals have adapted to their extreme environment. By understanding the unique locomotion strategies of Arctic wildlife, designers can gain inspiration for developing innovative solutions that are efficient, sustainable, and well-suited to harsh conditions. For example, the way polar bears swim through icy waters or how reindeer (*Rangifer tarandus*) navigate through deep snow can inform the design of vehicles or equipment that can operate effectively in remote northern areas. Exploring the mechanics of movement and their visual manifestations in the shapes of the limbs and bodies of Arctic wildlife can unlock new possibilities for designers to create functional, user-friendly, and environmentally friendly products and systems. In this section, we present the students’ analysis of bionic analogues—Arctic animals, and their potential applications in design. We will illustrate how these findings can be applied to the development of a conceptual oversnow vehicle for Yamal and briefly showcase another student’s project of an all-terrain vehicle for Chukotka.

**Figure 2:**
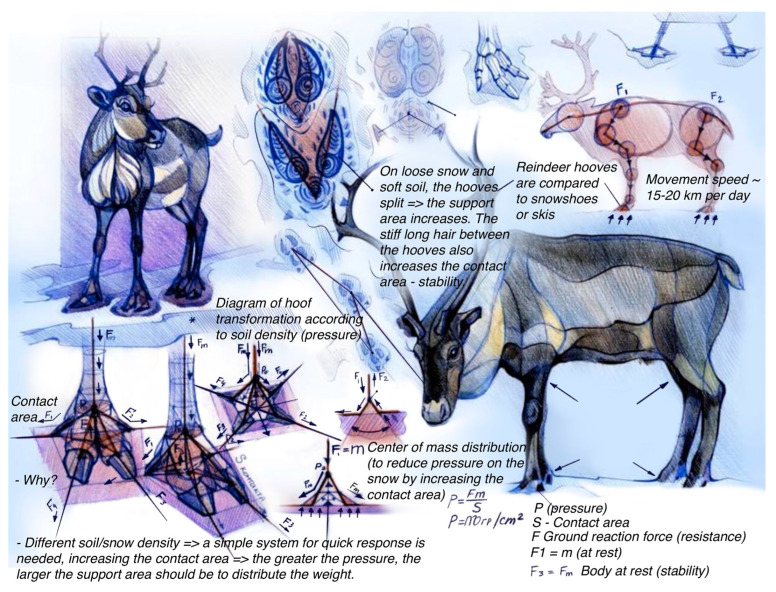
Student analyses of the biomorphology of reindeer. Source: E. Vasilieva.

Arctic animals have developed unique adaptations to move efficiently and silently across the local terrain. Many of them have a special mechanism in their limbs that allows them to quickly respond to changes in the bearing ground. For example, reindeer (Figure 2) have adaptable hooves that provide extra grip on wet surfaces in the summer with their soft foot pads, whereas in winter, their foot pads tighten, exposing the rim of their hoof for extra traction in snow and ice [32]. Reindeer bear their weight mainly on two digits, with their dewclaws aiding in navigating the snowy terrain. When walking, their foot pads and hoof capsules expand and flatten, cushioning the blow of each step, increasing the surface area of the support, and thus ensuring body stability [33]. Additionally, they have stiff hair in the interdigital space between their hooves, which is constantly under tension and bent along the radius, which helps break off ice formations on their hooves. 

**Figure 3:**
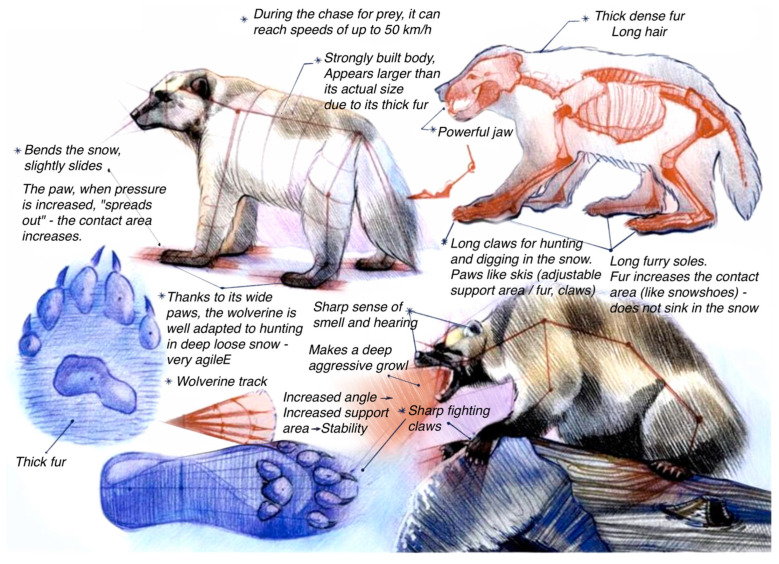
Student analyses of the biomorphology of wolverine. Source: E. Vasilieva.

A similar mechanism of a “snowshoe” foot for moving over unsupportive snow is also present in other Arctic animals, e.g., lynx, ptarmigan, wolverine (Figure 3), and polar bear [34,35,36]. 

**Figure 4:**
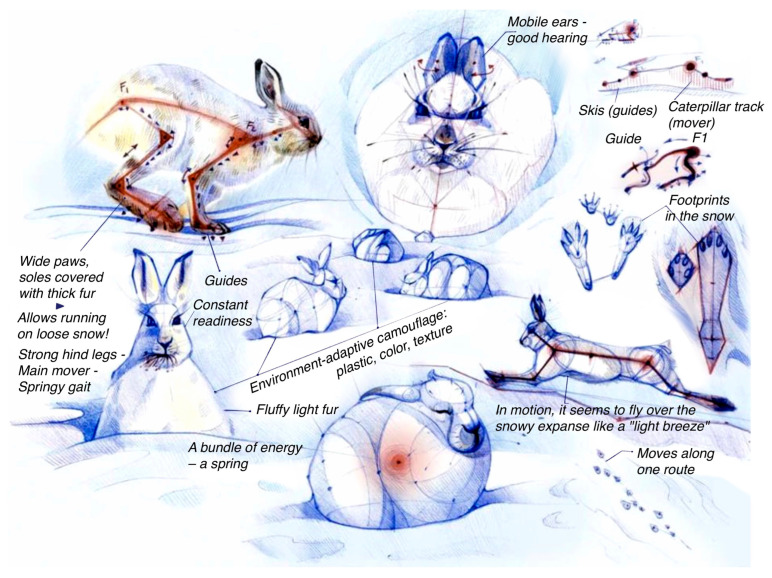
Student analyses of the biomorphology of the mountain hare. Source: E. Vasilieva.

Most Arctic animals use a “sliding smoothing” technique for movement instead of undermining. For example, the ptarmigan’s feet spread out in a circle in loose, marshy snow while decreasing the angle of the segment in dense snow. While running on snow, the ptarmigan slightly spreads its wings to reduce pressure on the ground and be able to glide. The mountain hare (Figure 4) adapts its movement technique to external conditions such as wet or loose snow. It is always ready to jump and represents a bundle of spring energy that transforms into a vector, i.e., a line when danger arises. Also, many Arctic animals have fur pads and an additional fur contour around their paws that ensure multi-level smooth distribution of load, resulting in a more silent gait.

Furthermore, despite the challenging environment, Arctic animals can move at impressive speeds. The average speed of Arctic animals ranges from 15 to 30 km/h, with some species such as wolverines and bears capable of developing speeds up to 50 km/h.

In addition to mechanical adaptations to changes in the bearing surface, many Arctic animals can analyze the characteristics of the terrain using their visual and auditory receptors. For example, hares have mobile ears that function as echolocators, while lynx have tufts on their ears that help them detect sounds. Mountain hares, polar foxes, and bears have sharp hearing and a sense of smell, which is aided by their low posture and wide field of view that allows them to see better and hear through the snow. These adaptations allow Arctic animals to navigate through their environment more effectively. 

Arctic mammals have adapted their vision to the extreme photic environment of the region. For instance, reindeer possess remarkable visual abilities that aid in their survival. Their visual range extends into the near ultraviolet (UV), allowing them to make greater use of the naturally UV-rich winter light [37]. They can also see predators at night, analyze the density of the snow based on its heat radiation, and locate food sources, such as lichen under the snow. In addition, most Arctic animals have panoramic vision with a wide field of view, enabling them to detect danger in their surroundings. For instance, hares have 360° vision, while reindeer have a field of view of 300–350°.

Overall, these adaptations demonstrate the ingenuity of nature and highlight the potential for bionics to inform technological innovations. For instance, the ability of certain animals to move efficiently on snow and ice could inform the design of vehicles that operate in similar environments. By incorporating features such as specialized treads or advanced sensors, these vehicles could traverse challenging terrains with greater ease and safety.

### 3.4. From Nature to Nomads—Bio-Inspired Design in Indigenous Arctic Transport 

For centuries, the Arctic indigenous peoples have developed and adapted mobility technologies and practices based on animal biomechanics. These technologies have demonstrated viable adaptations, such as gliding upon the snow/vegetation cover and moving at low velocities (typically between 10 to 20 km per hour), which allow for careful observation and reconnaissance of the land. Skis, snowshoes, and sleds are some of the examples of these technologies that have been passed down from generation to generation (for more research on these technologies, please refer to [38]).

**Figure 5:**
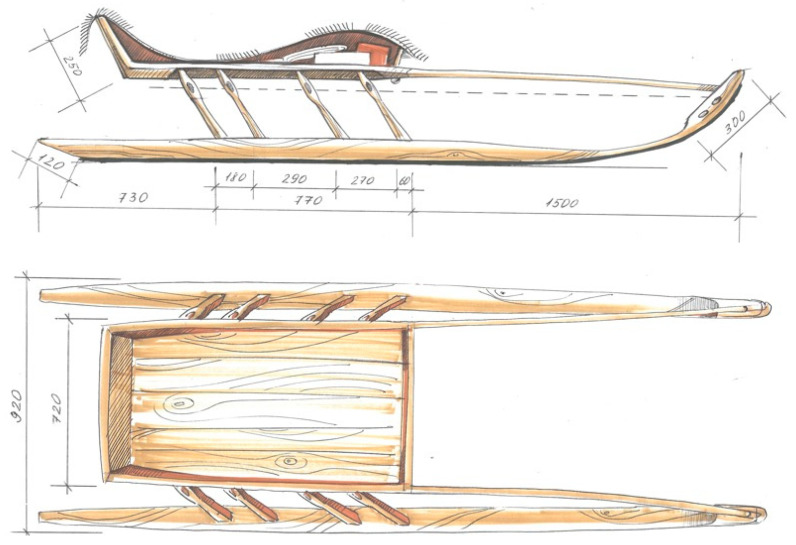
A male passenger sled. Field sketches by a student Ilya Polyanskikh.

In this section, we overview the sled, which is an integral component of a sled caravan. The sled serves as the primary mode of transportation for the nomadic people. There are summer and winter sleds, including passenger and cargo types, which make up a summer or winter caravan. More than a dozen varieties of sleds are used in Nenets caravans: lightweight passenger male (Figure 5), female; sacred; for firewood; for a boat; for fishing equipment; for tools and supplies; for clothes and hides; for food; for bedding, canopies and household clothes; for upper tent covers; for tent poles, cauldrons, and lower tent covers; for floorboards of the tent, mats made of rods, hearth sheet, and women’s shoes, etc. (for more information, see ([39], pp. 68–69)).

**Figure 6:**
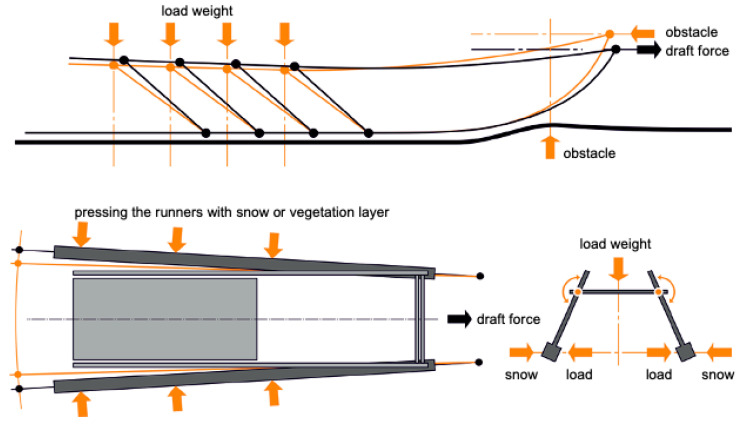
Features of the sled design. Top: load distribution during movement. Bottom: working of the sled structure during movement. A visual analysis of field data was conducted by Nikolai Garin. Drawing by Denis Kukanov. Source: ([39], p. 73).

The sled’s structural elasticity and mobility create an impression of a living creature, as evidenced by the names given to its various parts. For instance, the front of the sled is referred to as the “nose of the sled” (*khan’ pyya*), the back end as the “tail of the sled” (*khan’ yabtso*), and the runner as the “leg of the sled” (*khan’ nge*) ([39], p. 70). This intentional or unintentional practice by the Nenets reflects their desire to infuse their sleds with the qualities and characteristics of a living organism, which is considered a fundamental principle in their technology and design. “Living things” move organically and interact appropriately with nature and humans. Moreover, the Nenets sled exhibits a dynamic quality through the inclined positioning of its runners (Figure 6). Unlike conventional sleds with vertical runners, the runners of a Nenets sled are set at an angle, creating a visual impression of a creature poised for a leap or in a state of tension.

The slender-legged sled fearlessly negotiates both speed and weight, effortlessly navigating through snowy drifts, swamps, steep shores, and, when needed, even taking to the water and swimming alongside a reindeer team. The Nenets distinguish a graceful sled from a clumsy one, and in their evaluations, the qualities of “beauty” and “practicality” consistently merge into one notion.

The sled has its own lifespan, typically ranging from 3 to 10 years (although stories from tundra dwellers mention sleds that lasted over 30 years). To extend the longevity of the sled, an additional glider (*nyarma*) is attached to the runners. This strip, typically made of larch but nowadays also made of plastic or sheet metal, serves to protect the main runners. Also, larch is a high-density material that does not absorb water, making the runners especially suitable for traveling on sea ice, snow-covered ground in winter, as well as marshy terrains in spring and summer.

**Figure 7:**
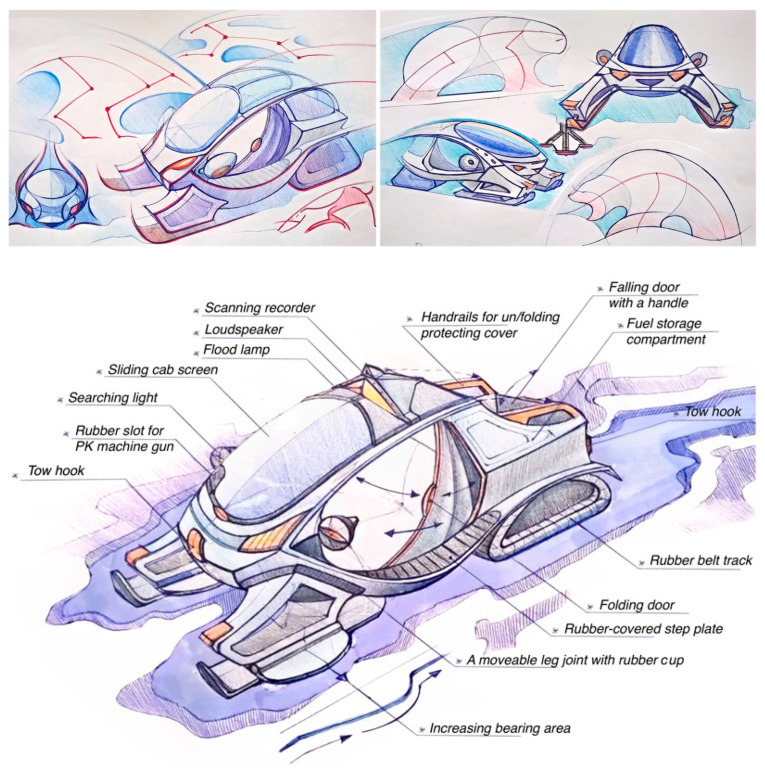
Biomimetic sketching inspired by a hare (**left and down**) and a wolverine (**right**). Source: E. Vasilieva.

The long and gradual curve of the front part of the runner helps to avoid sudden dynamic impacts that occur when encountering obstacles and traveling off-road (Figure 7 Top). This shape allows the sled to smoothly negotiate obstacles: when traversing deep and loose snow or slush, the runner does not cut through the snow cover but gradually compresses it to its maximum density beneath the heaviest part of the sled. This specific shape of the runner “encourages” the snow, regardless of its condition, to push the sled out to the surface. In other words, the combined force of the snow resistance during travel is directed upwards (similar to boats rising from the water during movement), ensuring stability and maneuverability.

When traveling over summer tundra or during the transitional seasons, the sled’s design operates similarly to when traveling over snow. The runners glide smoothly over the vegetation layer, initially pressing the warm upper parts of the cover against the cold humus layer. The latter, in turn, is pressed against the frozen layer, thus causing the track under the runner to slightly thaw and become moist and cultivating the soil beneath the runners. As a result, the heavy part of the runners (over which the load is placed) glides over the already-moistened vegetation layer.

The passage of the sled and entire caravan creates a cultivation effect and a favorable environment for plant growth due to the mixing of the layers. In their own way, reindeer do the same—not only do they eat the tops of grass and lichens, but they also cultivate and fertilize the soil beneath them. As a result of the prolonged impact of traditional transportation on the fragile vegetation cover of the tundra, areas or even entire strips of “roads” appear in places of constant contact, standing out against the yellow-green background of the tundra with their more lush greenery and abundant blooming cotton grass. These roads become more distinct or prominent as they are used more frequently, and they serve as reliable landmarks in the lowland tundra.

By studying animal adaptations and indigenous movement patterns, the students gained valuable insights into the essential features of systemic transportation solutions for the Arctic. For example, the student projects that focused on the Yamal Peninsula identified the following features:

(1) A safe average speed of 25 km/h, which allows one to watch the surroundings and detect changes, and a maximum speed of 60 km/h;
(2) Ground pressure between 10 and 25 g/cm^2^;
(3) A locomotion technique that involves the multi-level smooth distribution of weight, which prevents sinking or damaging the ground;
(4) A powerful and yet environmentally sound propulsion system, e.g., a “soft track”, and skis with good shock absorption and contact area adjustment using a “quick response” technology (a smart system that can quickly respond to variations in the bearing surface);
(5) An air gap in the skis or around the perimeter of the vehicle to maintain traction on marshy terrain, as well as protecting the support structure from moisture/ice;
(6) Low seating posture for stability;
(7) Maximum wide-angle view and powerful headlights and searchlights for patrolling and scanning the environment for danger;
(8) Smart “invisible” scanning of terrain using echolocators and infrared cameras to detect danger;
(9) Wind and snow protection via streamlined shaping;
(10) Maximum openness of the structure for interaction with the environment.

In addition, there are specific features that can be used to develop a “soft military presence” in the Arctic, such as landscape camouflage that is consistent with the environment (using appropriate colors, textures, and plasticity) and a biomorphic appearance that aids in friend-or-foe identification. 

**Figure 8:**
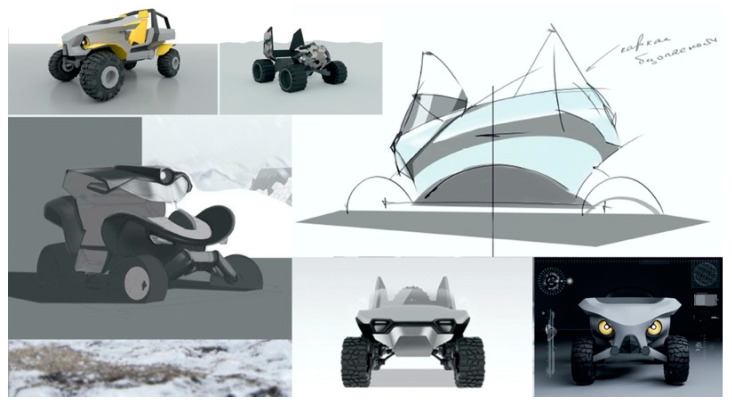
Biomimetic sketching inspired by tundra animals (owl and polar fox). Source: N. Klyusov.

As for the latter point, while working on the image, we thought it important to allow for the fact that border guarding takes place in an extreme environment and complex cultural space, i.e., alien to the outsider. During inevitable interactions between the border guards and the indigenous population, one should remember that the latter have their own semiotic range, which is different from the newcomer’s culture. Therefore, we searched for common reference points that would be positively or neutrally perceived by both sides, i.e., correspondent to the working environment and agreeing with the cultural context of a particular region. We decided to use widely known symbols and associations with the Arctic/Far North familiar to the newcomers and find an appropriate metaphor. Particularly, we relied on the Arctic fauna images to make use of both the expressive, instantly recognizable appearance of local animals and their unique biomechanical features that allow them to move through the complex terrain easily (Figure 7 and Figure 8).

#### Final Solution

The key design features of both vehicles are as follows:

- “side by side” seating of users;
- open body;
- bionic appearance.

The side-by-side position creates non-overlapping work zones and thus provides for task sharing: while the driver is being extremely attentive to the road, the gunner is on constant alert watching the area around. The open body (similar to the open seat of a musher on a reindeer sled) is responsive to the environment and allows quick evacuation and trace reading on the snow, while the low profile increases the stability of the vehicle. 

**Figure 9:**
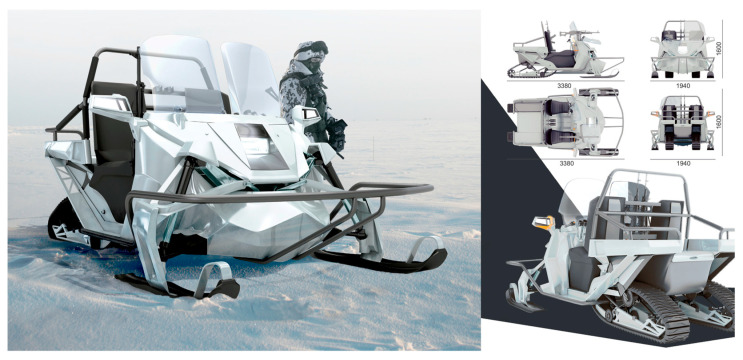
Snowmobile design for the Yamal Peninsula: A Master’s Degree Project by Ekaterina Vasilieva, assistant: Nikita Klyusov.

The vehicle presents a mixture of bionic traits (not a direct imitation of a particular animal) aimed at making it appealing/friendly looking and thus connecting it with the user and the environment of use. Also, in the snowmobile case (Figure 9), skis were designed using biomimetic principles based on the geometry of reindeer hooves. This design increased the contact area in loose snow and decreased it in dense snow, such as winter roads, granulated snow, and ice. However, it is important to note that the project’s goal was not to produce technical solutions directly inspired by the biology of Arctic animals. Instead, the goal was to conceptually justify the use of certain bio-adaptations and indicate the need for their further development in the context of snowmobile design.

**Figure 10:**
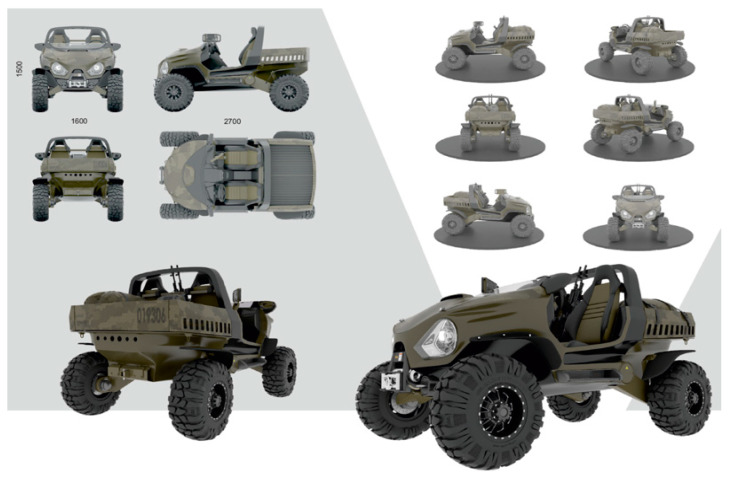
ATV design for the Chukchi Peninsula: A Master’s Degree project by Nikita Klyusov.

In the case of a wheeled all-terrain vehicle (ATV) (Figure 10), the bio-inspired idea of a changeable (terrain-dependent) wheel contact surface is realized by using low-pressure tires and tire pressure control. The driver can adjust the wheel contact surface area and the traction on a surface of any type.

## 4. Discussion and Conclusions

In this paper, we employed empirical data from two student projects centered on special-purpose vehicles for the Arctic to highlight the place-specificity of transport technology. Our main argument was to ensure user safety and sustainable functional readiness; the vehicle—besides being technologically efficient—must possess artistic and imaginative characteristics corresponding to the environmental/climatic, socio-cultural, and psychological peculiarities of use. 

Overall, the paper offers a new lens on developing special-purpose vehicles, especially on the often-overlooked visual appearance of these machines. The analysis and observations revealed some challenges associated with the “arctification” of the transport sector, particularly small-size all-terrain transport vehicles. These challenges are often ignored in most scholarly and public debates about Arctic transport, while there is a growing awareness that such means of transport should meet the requirements of both the changing environment and the user having to operate under the stress of adaptation to extreme conditions. 

Regarding further research into this topic, it is important to understand, predict, or even shape trends in the development of small-size vehicles for roadless and extreme climatic conditions by looking at different sites where such vehicles (and transport systems) are to be used. Today, locally appropriate technological developments are happening in real-world environments at the level of local user innovation activities [40,41,42]. In-depth fieldwork with such users will shed further light on technology’s role in how it can contribute to working and living in severe and challenging environments.

## Figures and Tables

**Table 1:** Standard machinery affected by cold temperatures. Source: Adapted from [24], original version in [25].

Temperature (°C)	Effects on Standard Machinery
−6	Internal combustion engines require pre-start engine heating
−10	Destruction of some standard metal dredge components
−15	High-carbon steel break; car batteries must be heated; first critical threshold for standard equipment
−20	Standard compressors with internal combustion engines cease to operate
−25 to −30	Unalloyed steels break; car-engine space, fuel tanks, and oil tanks must be insulated; frost-resistant rubber required
−30	Minimum temperature for use of any standard equipment
−35 to −40	Tin-alloyed steel components shatter; all compressors stop working; standard steels and structures rupture on a mass scale

## Data Availability

Due to legal and ethical restrictions, some of the data used in this study cannot be shared as they are contained in unpublished Doctoral and Master’s theses. However, a summary of the data can be provided upon request to the corresponding author.

## References

[1] Eikeland O.F., Bianchi F.M., Apostoleris H., Hansen M., Chiou Y.-C., Chiesa M. (2021). Predicting Energy Demand in Semi-Remote Arctic Locations. Energies.

[2] TMcDorman L., Schofield C. (2020). The Arctic Ocean unscrambled: Competing claims and boundary disputes. Research Handbook on Polar Law.

[3] Moe A., Stokke O.S. (2019). Asian Countries and Arctic Shipping: Policies, Interests and Footprints on Governance. Arct. Rev. Law Politics.

[4] Change W.F., Coates K.S., Holroyd C. (2020). Arctic Climate Change: Local Impacts, Global Consequences, and Policy Implications. The Palgrave Handbook of Arctic Policy and Politics.

[5] Alberque W., Schreer B. (2022). Finland, Sweden and NATO Membership. Survival.

[6] Sun D., Zoubir Y. (2012). From Hard Military Bases to Soft Military Presence: US Military Deployment in Iraq Reassessed. J. Middle East. Islam. Stud..

[7] Degang S. (2018). China’s Soft Military Presence in the Middle East.

[8] (2020). Ukaz Prezidenta RF ot 26 Oktyabrya 2020 g. N 645 «O Strategii Razvitiya Arkticheskoy Zony Rossiyskoy Fede-Ratsii I Obespecheniya Natsional’Noy Bezopasnosti NA Period Do 2035 Goda» Decree of the President of the Russian Federation of October 26, 2020 N 645 “Strategy for Developing the Russian Arctic Zone and Ensuring National Security through 2035. http://publication.pravo.gov.ru/document/View/0001202010260033?index=8.

[9] Kjellén J. (2022). The Russian Northern Fleet and the (Re)militarisation of the Arctic. Arct. Rev. Law Politics.

[10] Zhuravel V., Timoshenko D.S., Institute of Europe Russian Academy of Sciences (2022). The Russian Arctic, Sanctions Pressure and Geopolitical Instability. Arct. Rev. Law Politics.

[11] Dobretsov R., Porshnev G., Uvakin D. (2018). Performance improvement of Arctic tracked vehicles. MATEC Web Conf..

[12] Galeotti M., Hook A. (2020). Combat vehicles of Russia’s Special Forces: Spetsnaz, Airborne, Arctic and Interior Troops.

[13] Usenyuk-Kravchuk S., Akimenko D., Garin N., Mietteinen S. (2020). Arctic Design: Basic Concepts and Practice of Implementation. RELATE NORTH: Tradition and Innovation in Art and Design Education.

[14] Garin N., Usenyuk S., Kukanov D., Gostyaeva M., Konkova Y., Rogova A. (2017). Arctic Design School. Album-Monograph.

[15] He L., Chen W. Usage context-based choice modeling for hybrid electric vehicles. Proceedings of the DS 68-10: The 18th International Conference on Engineering Design (ICED11).

[16] Fayemi E.I., Maranzana N., Aoussat A., Chekchak T., Bersano G. Modeling biological systems to facilitate their selection during a bio-inspired design process. Proceedings of the 20th International Conference on Engineering Design (ICED15).

[17] Lenau T. Biomimetics as a design methodology—Possibilities and challenges. Proceedings of the ICED’09.

[18] Lenau T. Do biomimetic students think outside the box?. Proceedings of the DS 87-4: 21st International Conference on Engineering Design (ICED 17).

[19] Lenau T., Keshwani S., Chakrabarti A., Ahmed-Kristensen S. Biocards and Level of Abstraction. Proceedings of the 20th International Conference on Engineering Design (ICED15).

[20] Larin S.I. (2001). Geografiya Yamalo-Nenetskogo Avtonomnogo Okruga: Uchebnoe Posobie Geography of the Yamalo-Nenets Autonomous Okrug: Textbook.

[21] Andreev V. (1986). Biologicheskiye Statsionary Sovetskogo Severa [Biological Stations of the Soviet North].

[22] Golubchikov Y.N. (2003). Geografija Chukotskogo Avtonomnogo Okruga. Uchebnoe Posobie Dlya Srednikh Shkol [Geography of the Chukotka Autonomous Okrug. Textbook for Secondary Schools].

[23] Dibbern J.S. (1977). Soviet and Japanese oversnow vehicles. J. Terramechanics.

[24] Mote V.L., RJensen G., Shabad T., Wright A.W. (1983). Environmental Constraints to the Economic Development of Siberia. Soviet Natural Resources in the World Economy.

[25] Dogayev Y. (1969). Ekonomicheskaya Effektivnost Novoi Tekhniki na Severe [The Economic Efficiency of New Technology in the North].

[26] Bulygina O.N., Razuvaev V.N., Korshunova N.N. (2009). Changes in snow cover over Northern Eurasia in the last few decades. Environ. Res. Lett..

[27] Krupnik I. (2011). ‘How many Eskimo words for ice?’ Collecting Inuit sea ice terminologies in the International Polar Year 2007–2008. Can. Geogr. Le Géographe Can..

[28] Sturm M., Holmgren J., Liston G.E. (1995). A Seasonal Snow Cover Classification System for Local to Global Applications. J. Clim..

[29] Fedenko E.M., Pevnev N.G. (2018). Improving the crew car vehicles safety operated in the conditions of the Far North. Obrazovaniye. Transport. Innovatsii. Stroitel’stv.

[30] Saenko V. (2017). Experience of investigation of search-rescue works under the tundra conditions in Taymyr region. Aviation Rescue Technologies in Providing an Integrated Security System in the Arctic Region: Collection of Research Papers of the VI Forum of EMERCOM, 13–16 July 2017.

[31] Voronkov Y. (2017). Vliyaniye protsessa gumanizatsii usloviy truda na resheniye sotsial’no-trudovykh problem voditeley severnogo regiona Rossii [Influence of the process of humanization of working conditions on the solution of social and labour problems of drivers in the northern region of Russia]. Bezopasnost’ Gorodskoy Sredy [Urban Safety].

[32] Wells J., Watson K., Daniel C.R., Brodell R.T., Nahar V.K. (2022). Holiday Reindeer Trivia for Physicians Who Like Hair and Nails. Ski. Appendage Disord.

[33] Hull E., Semeniuk M., Puolakka H.-L., Kynkäänniemi S.-M., Niinimäki S. (2021). Tendons and ligaments of the Rangifer tarandus metapodial and hoof. Polar. Biol..

[34] Mármol-Guijarro A., Nudds R., Folkow L., Sellers W., Falkingham P., Codd J. (2021). The Influence of Snow Properties on Speed and Gait Choice in the Svalbard Rock Ptarmigan (*Lagopus muta hyperborea*). Integr. Org. Biol..

[35] Grevtsev V.I. (2007). Osobennosti Ekologii Rosomakhi i Yeye rol’ v Okhotnich’yem Khozyaystve [The Peculiarities of Wolverine ecology and its Role in Hunting Economics]. Sovrem. Probl. Prir. Okhotovedeniya I Zverovod..

[36] Abourachid A., Gasc J.-P., Renous S. (1997). Kinematic Analysis of the Locomotion of the Polar Bear (Ursus Maritimus, Phipps, 1774) in Natural and Experimental Conditions. Neth. J. Zool..

[37] Hogg C., Neveu M., Stokkan K.A., Folkow L., Cottrill P., Douglas R., Hunt D.M., Jeffery G. (2011). Arctic reindeer extend their visual range into the ultraviolet. J. Exp. Biol..

[38] Garin N., Kravchuk S. (2018). Sliding Vehicles for the Russian North: The History of Ideas (Album-Monograph).

[39] Golovnev A., Garin N., Kukanov D. (2016). Reindeer Herders of Yamal (Research Materials for the Atlas of Nomadic Technologies).

[40] Hyysalo S., Usenyuk S. (2015). The user dominated technology era: Dynamics of dispersed peer-innovation. Res. Policy.

[41] Usenyuk S., Hyysalo S., Whalen J. (2016). Proximal Design: Users as Designers of Mobility in the Russian North. Technol. Cult..

[42] Usenyuk-Kravchuk S., Hyysalo S., Raeva A. (2022). Local adequacy as a design strategy in place-based making. CoDesign.

